# Correction: A highly X-ray sensitive iridium prodrug for visualized tumor radiochemotherapy

**DOI:** 10.1039/d1sc90029c

**Published:** 2021-02-15

**Authors:** Zhennan Zhao, Pan Gao, Li Ma, Tianfeng Chen

**Affiliations:** Department of Chemistry, Jinan University Guangzhou 510632 China tchentf@jnu.edu.cn chem-mali@foxmail.com

## Abstract

Correction for ’A highly X-ray sensitive iridium prodrug for visualized tumor radiochemotherapy’ by Zhennan Zhao *et al.*, *Chem. Sci.*, 2020, **11**, 3780–3789, DOI: 10.1039/D0SC00862A

The authors regret errors in Fig. 5a in the original version of this *Chemical Science* article, specifically the 0 h and 24 h images in the top row (the Ir–CH treated group) and the 12 h and 36 h images in the bottom row (the Ir–NB treated group) which were used in Fig. 5a in error. The authors therefore wish to replace Fig. 5a and b with data from parallel experiments to ensure uniform intensity units in the colour bar. The correct version of Fig. 5 is shown below. This does not affect any of the discussions or conclusions reported in the article.

**Fig. 5:**
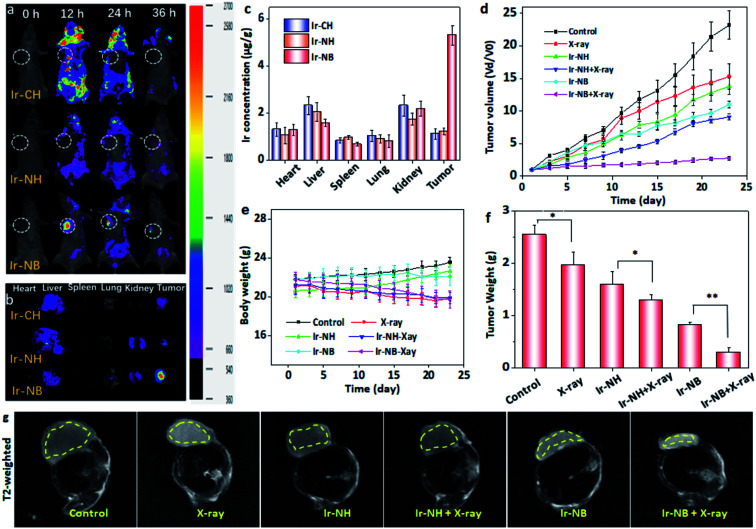
Image-guided cancer radiochemotherapy of the Ir(iii) complexes in xenograft mouse models. (a) Luminescence images of xenograft nude mice after intravenous injection of the Ir complexes (2 μmol kg^−1^) at different time points. The tumor site was highlighted by the dashed circle. (b) The major organs and tumors were acquired from the sacrificed mice and imaged 36 h after injection of the Ir(iii) complexes. (c) The bio-distribution of the Ir complexes was determined by the Ir content in major organs and tumor sites 36 h after injection. The recorded (d) tumor volume, (e) body weight and (f) tumor weight of mice with A549 xenograft after various treatment for 23 days. Each value represents means ± SD (*n* = 3). Bars with different characteristics are statistically different levels *P < 0.05, **P < 0.01. (g) T_2_-weighted MR images of A549 tumor-bearing mice after different treatments for 23 days. The tumor sites are in the back region and circled by dashed lines.

The Royal Society of Chemistry apologises for these errors and any consequent inconvenience to authors and readers.

